# Stable isotope variations of dew under three different climates

**DOI:** 10.1038/s41597-022-01151-6

**Published:** 2022-02-14

**Authors:** Chao Tian, Kun Du, Lixin Wang, Xiao Zhang, Fadong Li, Wenzhe Jiao, Daniel Beysens, Kudzai Farai Kaseke, Marie-Gabrielle Medici

**Affiliations:** 1 Key Laboratory of Ecosystem Network Observation and Modeling, Institute of Geographic Sciences and Natural Resources Research, Chinese Academy of Sciences, Beijing, 100101 China; 2 Department of Earth Sciences, Indiana University-Purdue University Indianapolis (IUPUI), Indianapolis, IN 46202 USA; 3 Shandong Yucheng Agro-ecosystem National Observation and Research Station, Ministry of Science and Technology, Yucheng, 251200 China; 4 School of Resources and Environment, LinYi University, Linyi, 276000 China; 5 College of Resources and Environment, University of Chinese Academy of Sciences, Beijing, 100049 China; 6 Key Laboratory of Geospatial Technology for the Middle and Lower Yellow River Regions, Ministry of Education. College of Environment and Planning, Henan University, Jinming Avenue, Kaifeng, 475004 China; 7 Physique et Mécanique des Milieux Hétérogènes, CNRS, ESPCI, PSL Research University, Sorbonne Université, Sorbonne Paris Cité, 75005 Paris, France; 8 OPUR, 2 rue Verderet, 75016 Paris, France; 9 Earth Research Institute, University of California, Santa Barbara, CA 93106 USA; 10 LPMC, Université de Nice, CNRS-UMR 7336, 06108 Nice Cedex 2, France

**Keywords:** Hydrology, Environmental sciences

## Abstract

As a supplementary or the only water source in dry regions, dew plays a critical role in the survival of organisms. The new hydrological tracer ^17^O-excess, with almost sole dependence on relative humidity, provides a new way to distinguish the evaporation processes and reconstruct the paleoclimate. Up to now, there is no published daily dew isotope record on δ^2^H, δ^18^O, δ^17^O, d-excess, and ^17^O-excess. Here, we collected daily dew between July 2014 and April 2018 from three distinct climatic regions (i.e., Gobabeb in the central Namib Desert with desert climate, Nice in France with Mediterranean climate, and Indianapolis in the central United States with humid continental climate). The δ^2^H, δ^18^O, and δ^17^O of dew were simultaneously analyzed using a Triple Water Vapor Isotope Analyzer based on Off-Axis Integrated Cavity Output Spectroscopy technique, and then d-excess and ^17^O-excess were calculated. This report presents daily dew isotope dataset under three climatic regions. It is useful for researchers to use it as a reference when studying global dew dynamics and dew formation mechanisms.

## Abstract (summary)

**Table:** 

Measurement(s)	stable isotope variation • dew
Technology Type(s)	water vapour isotope analysis
Factor Type(s)	climate • temporal interval
Sample Characteristic - Environment	desert climate • Mediterranean climate • humid continental climate
Sample Characteristic - Location	Namib Desert • Nice • Indianapolis

Machine-accessible metadata file describing the reported data: 10.6084/m9.figshare.19070114

## Background & Summary

Global warming has increased the demand of moisture in the local atmosphere, leading to a decrease in precipitation over many regions, both of which could contribute to drought^1,2^. Water vapor can condense as dew on a surface where radiation cools below the dew point temperature^3,4^. Dew, as a significant source of non-rainfall water, is considered a vital water source due to its considerable contribution for the surface water balance, especially in semiarid and arid regions^3,5–8^. The annual dew amounts account for 9% to 23% of rainfall in arid regions^7,9^. Dew could be used as an alternative source of water during dry season of tropical islands^10^. Dew is beneficial to the survival, growth and development of the plants in arid regions or during droughts, such as bringing nocturnal moisture^11–13^ and being directly absorbed and utilized by leaves through plant stomata or special physical features (e.g., aerial plants)^14–16^. Thus, dew could increase net photosynthate accumulation in leaves^17^ and improve plant water use efficiency^16,18^. Dew is also involved in the chemical processes in the atmosphere, such as the diurnal (and nocturnal) cycle of nitrites oxides^3,19^. Dew frequency decreased by 5.2 days per decade from 1961 to 2010 in China due primarily to near-surface warming and associated decreases in relative humidity (RH)^11^. Furthermore, the decreasing rate of dew frequency in arid regions (50%) is higher than that in semi-humid and humid regions in China (40% and 28%)^11^. Therefore, dew in different regions have different trends with the global climate change, and dew characteristics under different climate regions are needed to better predict future changes in dew dynamics.

δ^2^H and δ^18^O are natural and traditional hydrological tracers, and play an important role to trace different hydrometeorological processes associated with different types of waters (e.g., rainfall, snowfall, dew, fog, surface water, plant water, and ice core)^20–24^. Two types of mass-dependent fractionation process, equilibrium fractionation and kinetic fractionation, is the fundamental cause of isotope differences during water phase change^25–28^. They are mainly determined by the saturation vapor pressure and the diffusion rate of different isotopes, respectively^29–31^.

^17^O-excess (^17^O-excess = ln (δ^17^O + 1) − 0.528 x ln (δ^18^O + 1)), a newer hydrological tracer, became available to provide additional constraints about moisture transport, rainout, and evaporation to probe hydrological and meteorological processes^32,33^. Compared with the conventional isotopes depending on both temperature and RH, the ^17^O-excess is mainly sensitive to the RH between 10°C to 45°C^34,35^. The RH dependence is also confirmed by field experimental observations such as monsoon precipitation and leaf water in Africa^36,37^, precipitation on a subtropical island^38^, surface water across the Pacific Northwest, USA^39^, and ice cores in coastal East Antarctica^40^. The relationship between δ′^18^O and δ′^17^O (i.e., the slope of 1000 x ln (δ^18^O + 1) and 1000 x ln (δ^17^O + 1))^41^ can be used to better reveal tap water and precipitation formation mechanisms^42^, differentiate drought types (e.g., synoptic drought vs. local drought)^43^, and distinguish different types of condensation (e.g., fog vs. dew) in the Namib Desert^44^. Furthermore, it is an effective method to infer the different water evaporation processes experiencing equilibrium fractionation or kinetic fractionation using the relationships between ^17^O-excess and δ′^18^O (or d-excess) (e.g., laboratory model test, precipitation, and natural water bodies (river, channels, wells, springs, groundwater, lake and ponds))^32,34,36,45–50^.

To the best of our knowledge, there is no daily dew isotope dataset including ^17^O-excess publicly available. Here, we provide daily dew isotope dataset (δ^2^H, δ^18^O, δ^17^O, d-excess, and ^17^O-excess) under three different climatic regions including Gobabeb-Namib Research Institute (hereafter Gobabeb) in the central Namib Desert with desert climate, Nice in France with Mediterranean climate, and Indianapolis in the central United States with humid continental climate collected between July 2014 to April 2018. Our previous studies have described the operating procedures of Triple Water Vapor Isotope Analyzer (T-WVIA-45-EP; Los Gatos Research Inc. (LGR), Mountain View, CA, USA) based on Off-Axis Integrated Cavity Output Spectroscopy (OA-ICOS) technique^46,51^, as well as the detailed description of ^17^O-excess quality control method. The dew isotopic variations have been published in Tian *et al*.^52^. This first publicly available daily dew isotope dataset were presented to fill the gap in global non-rainfall water isotope datasets, especially for ^17^O-excess. This would provide data support for scientists to study the global dew distribution characteristics and formation mechanism under climate change.

## Methods

### Sample collections

The daily dew samples were collected in three different climatic regions including 22 samples in Gobabeb (23.55° S, 15.04° E; 405 m above sea level) with desert climate from July 2014 to June 2017, 23 samples in Nice (43.74° N, 7.27° E; 310 m above sea level) with Mediterranean climate from December 2017 to April 2018, and 69 samples in Indianapolis (39.88° N, 86.27° W; 258 m above sea level) with humid continental climate from January 2017 to October 2017. The detail site meteorological information has been descripted by Tian *et al*.^52^. In short, the mean annual temperature (hereafter MAT), mean annual relative humidity (hereafter MARH), and mean annual precipitation amount (hereafter MAP) in Gobabeb is 21.1°C, 50%, and < 20 mm, respectively. The MAT, MARH, and MAP in Nice is 16.0°C, 78%, and 733 mm, respectively. The MAT, MARH, and MAP in Indianapolis is 10.2°C, 69%, and 953 mm, respectively. For Gobabeb, there are concurrent fog and dew collectors installed at the same location, collected water samples were considered dew when water samples appear in dew collector and no samples in the fog collector. For Nice and Indianapolis, fog is rarely seen, dew samples were separated from fog or light rain based on local meteorological information and visual observation of the collecting personnel. All of daily dew samples were collected before dawn to minimize evaporation effects on isotopes, and they were stored in 15 ml sealed glass vials in Gobabeb and Indianapolis or polyethylene bottles for the dew samples in Nice. All of the 114 dew samples were delivered to the IUPUI (Indiana University-Purdue University Indianapolis) Ecohydrology Lab to measure δ^2^H, δ^18^O and δ^17^O. Here, the detailed daily dew isotopic variations were reported especially for ^17^O-excess values.

### Isotope measurements and ^17^O-excess data processing

The isotopic variations was measured using a Triple Water Vapor Isotope Analyzer (T-WVIA-45-EP, Los Gatos Research Inc. (LGR), Mountain View, CA, USA; preheated to 50°C) coupled to a Water Vapor Isotope Standard Source (WVISS, LGR, Mountain View, CA, USA; preheated to 80°C)^53^. The detailed operation has been described by Tian *et al*.^42,51^. To ensure the accuracy of the T-WVIA performance, LGR#1 to LGR#5 as five working standards from LGR were analyzed after every five samples. The known δ^2^H from LGR#1 to LGR#5 is −154.0‰, −123.7‰, −97.3‰, −51.6‰, and −9.2‰, respectively. The known δ^18^O from LGR#1 to LGR#5 is −19.49‰, −16.24‰, −13.39‰, −7.94‰, and −2.69‰, respectively. The known δ^17^O from LGR#1 to LGR#5 is −10.30‰, −8.56‰, −7.06‰, −4.17‰, and −1.39‰, respectively. Additionally, to reduce differences between laboratories, all of the isotope ratios were normalized using Vienna Standard Mean Ocean Water (VSMOW) and Standard Light Antarctic Precipitation (SLAP) once a day^54,55^. The δ^2^H, δ^18^O, and δ^17^O of SLAP are −427.5‰, −55.5‰, and −29.6986‰, respectively^55,56^. Furthermore, ^17^O-excess with small order of magnitude, need to quality control using raw δ^17^O and δ^18^O values to obtain accurate value. The detailed quality-control steps could be found in our previous studies^46,51^. To summarize, each individual data point was checked using two types of quality control filters. Firstly, because regression coefficient λ (λ = ln (δ^17^O + 1)/ln (δ^18^O + 1)) is the same as mass-dependent fractionation coefficient (θ) during liquid-vapor equilibrium and in water vapor diffusion in air^34,57^, and theoretically the θ was found to be 0.511 ± 0.005 for kinetic transport effects^57^ and 0.529 ± 0.001 for equilibrium effects^34^. Individual data points with regression coefficient λ outside the range of 0.506 and 0.530 were removed. Secondly, ^17^O-excess values that exceed the range of −100 to + 100 per meg were removed, which exceed the range of observed global precipitation ^17^O-excess values^34,36,47,57,58^. The final ^17^O-excess value of each dew sample was the mean value of all the individual data points meet the above the two conditions.

## Data Records

**Fig. 1:**
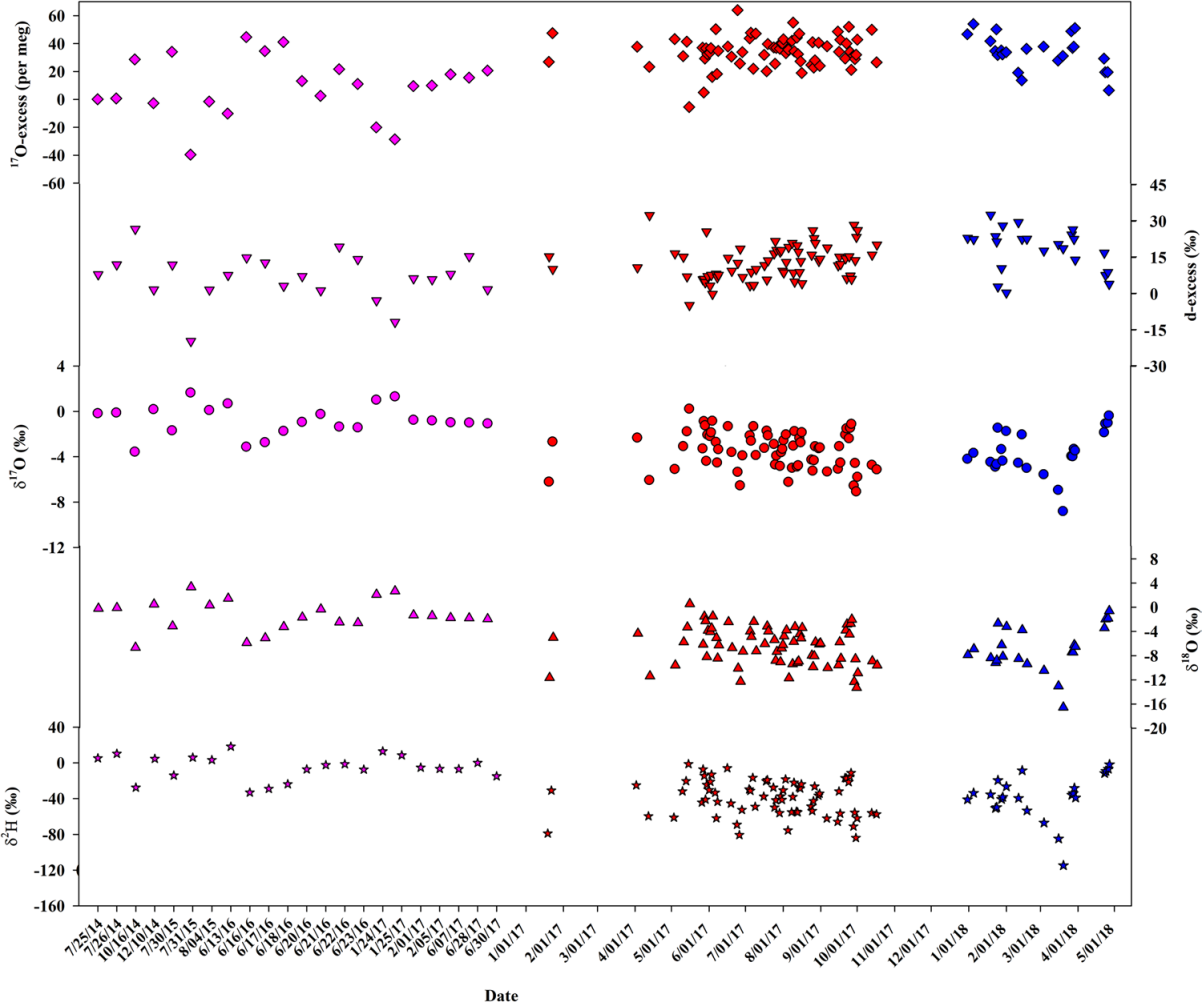
Dew stable isotope variations at Gobabeb (purple colors), Nice (blue colors), and Indianapolis (red colors). From top to bottom: ^17^O-excess, d-excess, δ^17^O, δ^18^O, and δ^2^H modified from Tian *et al*.^52^.

**Fig. 2:**
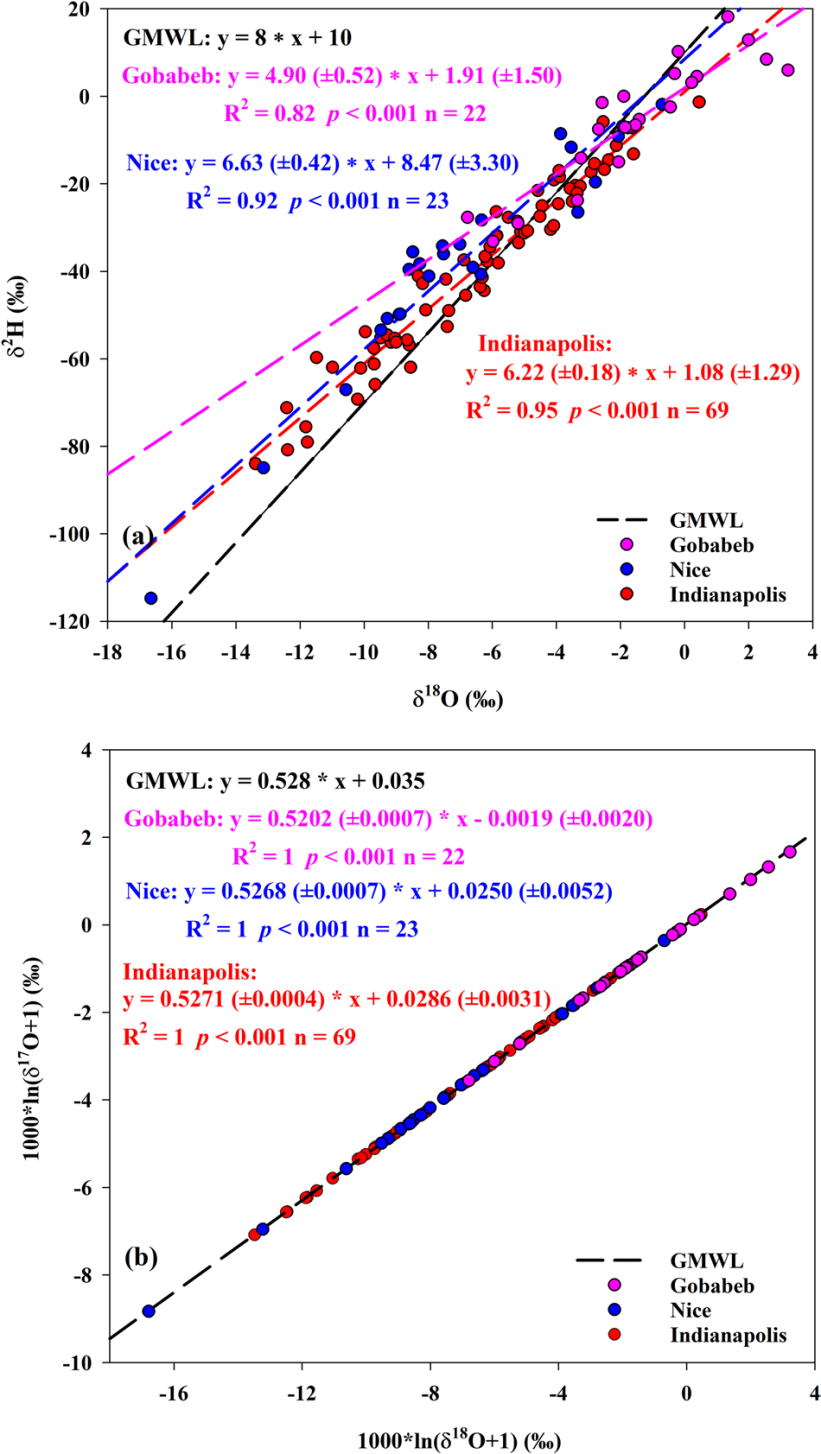
The relationships between δ^18^O and δ^2^H (**a**), as well as between δ′^18^O and δ′^17^O based on daily dew at Gobabeb, Nice, and Indianapolis.

Daily dew isotope database is archived in PANGAEA in a single table including 114 rows and 13 columns^59^. Each row presents a daily dew event at one site. Each column corresponds to the geographic location information (including latitude, longitude, and elevation), isotope variables including three measured individual stable isotopes (δ^2^H, δ^18^O, and δ^17^O) and two calculated second-order isotopic variables (d-excess and ^17^O-excess), and three meteorological information including temperature, RH, and VPD. A summary of the dew from July 2014 to April 2018 under three different climatic regions (Gobabeb, Nice, and Indianapolis) is presented in Table 1. The database spanned a large gap over 67.29° in latitude (from 23.55°S to 43.74°N) and 101.3° in longitude (from 86.27°W to 15.04°E). However, the difference in elevation was relatively small ranging from 258 m to 405 m. The meteorological factors show significant difference ranging from 1.39 °C to 21.36 °C for temperature, from 35.3% to 99.8% for RH, and from 0.2 hPa to 52.7 hPa for VPD (Table 1). Figure 1 depicts the distribution of daily dew stable isotopes in the three sites, which was modified from our previous study^52^. As for the dew in Gobabeb, the δ^2^H values varied from −33.21‰ to 18.17‰ with a mean value of −5.11 ± 14.03‰. The δ^18^O values varied from −6.77‰ to 3.23‰ with a mean value of −1.43 ± 2.59‰. The δ^17^O values varied from −3.55‰ to 1.66‰ with a mean value of −0.75 ± 1.35‰. The d-excess values varied from −19.9‰ to 26.5‰ with a mean value of 6.3 ± 10.0‰. The ^17^O-excess values varied from −39 to 45 per meg with a mean value of 9 ± 22 per meg. As for the dew in Nice, the δ^2^H values varied from −114.77‰ to −1.90‰ with a mean value of −37.92 ± 25.91‰. The δ^18^O values varied from −16.65‰ to −0.70‰ with a mean value of −7.00 ± 3.75‰. The δ^17^O values varied from −8.79‰ to −0.36‰ with a mean value of −3.67 ± 1.99‰. The d-excess values varied from 0.1‰ to 32.3‰ with a mean value of 18.1 ± 8.8‰. The ^17^O-excess values varied from 7 to 54 per meg with a mean value of 34 ± 12 per meg. As for the dew in Indianapolis, the δ^2^H values varied from −83.99‰ to −1.34‰ with a mean value of −39.38 ± 19.81‰. The δ^18^O values varied from −13.39‰ to 0.46‰ with a mean value of −6.51 ± 3.10‰. The δ^17^O values varied from −7.06‰ to 0.24‰ with a mean value of −3.41 ± 1.64‰. The d-excess values varied from −5.0‰ to 32.1‰ with a mean value of 12.7 ± 7.2‰. The ^17^O-excess values varied from −5 to 64 per meg with a mean value of 35 ± 11 per meg. Linear least-squares fitting was utilized to determine the slope and intercept of the dew line in the three sites. The numbers in the parenthesis were standard errors of the estimates. The dew line in Gobabeb between δ^18^O and δ^2^H was δ^2^H = 4.90 ( ± 0.52) x δ^18^O + 1.91 ( ± 1.50) (R^2^ = 0.82, *p* < 0.001), which had the lowest slope and intercept than those in Nice and Indianapolis (Fig. 2a). The slope and intercept in Nice were 6.63 and 8.47, and those in Indianapolis were 6.22 and 1.08. All of the three dew lines were far from the Global Meteoric Water Line (GMWL, δ^2^H = 8 x δ^18^O + 10). The dew line in Gobabeb between δ′^18^O and δ′^17^O was δ′^17^O = 0.5202 ( ± 0.0007) x δ′^18^O − 0.0019 ( ± 0.0020) (R^2^ = 1, *p* < 0.001), far from the GMWL for oxygen (δ′^17^O = 0.528 x δ′^18^O + 0.035, normalized to the VSMOW-SLAP scale^47,58^ (Fig. 2b). The slope and intercept of the dew line between δ′^18^O and δ′^17^O in Nice (0.5268 and 0.0250) was similar with those in Indianapolis (0.5271 and 0.0286), both of which were close to the GMWL.

**Table 1:** Summary of the daily dew record at Gobabeb (from July 2014 to June 2017), Nice (from December 2017 to April 2018), and Indianapolis (from January 2017 to October 2017).

Site (Country)	Latitude (°)	Longitude (^o^)	Elevation (m, a.s.l)	Köppen climate classification		δ^2^H (‰)	δ^18^O (‰)	δ^17^O (‰)	d-excess (‰)	^17^O-excess (per meg)	Temperature (^o^C)	Relative Humidity (%)	Vapor Pressure Deficit (hPa)
Gobabeb (Namiba)	−23.55	15.04	405	Desert climate	Minimum	−33.21	−6.77	−3.55	−19.9	−39	3.53	35.3	1.3
					Maximum	18.17	3.23	1.66	26.5	45	16.85	98.3	52.7
					Mean	−5.11	−1.43	−0.75	6.3	9	11.83	77.6	17.1
					Standard deviation	14.03	2.59	1.35	10.0	22	3.67	17.8	14.2
Nice (France)	43.74	7.27	310	Mediterranean climate	Minimum	−114.77	−16.65	−8.79	0.1	7	3.55	55.1	4.6
					Maximum	−1.90	−0.70	−0.36	32.3	54	15.25	94.2	31.0
					Mean	−37.92	−7.00	−3.67	18.1	34	9.06	80.4	14.0
					Standard deviation	25.91	3.75	1.99	8.8	12	3.01	9.6	6.6
Indianapolis (United State)	39.88	−86.27	258	Humid continental climate	Minimum	−83.99	−13.39	−7.06	−5.0	−5	1.39	65.9	0.2
					Maximum	−1.34	0.46	0.24	32.1	64	21.36	99.8	27.1
					Mean	−39.38	−6.51	−3.41	12.7	35	13.91	91.9	6.2
					Standard deviation	19.81	3.10	1.64	7.2	11	4.39	6.5	5.0

## Technical Validation

The precisions of different isotopic variables for two international standards (SLAP and Greenland Ice Sheet Precipitation) and the five working standards (LGR#1 to LGR#5) have been measured in our previous studies, which was <0.80‰, <0.06‰, <0.03‰, and <12 per meg for δ^2^H, δ^18^O, δ^17^O, and ^17^O-excess, respectively^42,51^. The precisions of our OA-ICOS measurements are within the range of analyzers using the same technique (0.07‰, 0.05‰, and 10 to 18 per meg for δ^18^O, δ^17^O, and ^17^O-excess, respectively)^60^. Comparing with the Cavity Ring Down Spectroscopy (CRDS) technique, our precisions are comparable with previous studies (<0.98‰, <0.10‰, <0.10‰, and <10 per meg for δ^2^H, δ^18^O, δ^17^O, and ^17^O-excess, respectively)^54,61^. Our precisions are also acceptable compared with the traditional Isotope Ratio Mass Spectrometry (IRMS) technique (<0.7‰, <0.3‰, <0.05‰, and <16 per meg for δ^2^H, δ^18^O, δ^17^O, and ^17^O-excess, respectively)^47,55,58,62^.

## Supplementary information

Data file 1

## Data Availability

No custom code was used to generate or process the data.
